# The methyltransferase-like proteins as core regulators of nucleic acid modifications and post-translation modification of proteins in disease pathogenesis and therapeutic implications

**DOI:** 10.1186/s40364-025-00858-z

**Published:** 2025-11-05

**Authors:** Shenyuqi Wu, Duancheng Guo, Xichun Hu, Mengdi Yang

**Affiliations:** 1 Department of Medical Oncology, Fudan University Shanghai Cancer Center, Shanghai, 200032 People’s Republic of China; 2 Department of Oncology, Shanghai Medical College, Fudan University, Shanghai, 200032 People’s Republic of China

**Keywords:** METTLs, Nucleic acid modifications, Post-translational modifications cancer biology, Metabolic

## Abstract

The methyltransferase-like (METTL) family members are the central ‘writers’ of epitranscriptome modifications, catalyzing N6-methyladenosine (m^6^A), N7-methylguanosine (m^7^G), 3-methylcytosine (m^3^C) and other chemical markers that modify DNA, RNA, and proteins (both histones and non-histone proteins) to dynamically regulate gene expression. The METTL family is distinguished by structural diversity, substrate specificity and multifaceted roles in epigenetic regulation. Dysregulation of METTL proteins has been demonstrated to disrupt RNA stability, translational efficiency and signaling pathways, which has been associated with tumorigenesis, neurodegeneration and immune dysfunction. At present, there are still limitations in the knowledge of the cooperative networks among METTL members and with other major signaling pathways. The objective of the present study is to elucidate the regulatory mechanisms mediated by METTL across different levels, laying the groundwork for subsequent development of precision therapies targeting phenotypic enzyme modifications. This review comprehensively delineates the structural characteristics and molecular functions of METTLs, their cooperative interactions, and their pathophysiological regulatory networks organized by signaling pathways rather than disease categories. We evaluate their diagnostic potential as biomarkers and their therapeutic implications, with particular focus on emerging METTL inhibitors that have entered clinical trials. By systematically exploring the mechanisms behind their context-dependent functions and analyzing their potential for clinical translation, we provide a foundation for precision therapies targeting these core regulators of nucleic acid and protein methylation.

## Background

Nucleic Acid Modifications and Protein post-translational modifications (PTMs) have become the most exciting frontier in the field of life sciences after genomics. This field has overturned traditional cognition: RNA is not only a transmitter of genetic information, but its chemical modifications also constitute a “second layer of code” for dynamic regulation, precisely controlling the spatiotemporal specificity of gene expression [1, 2]. As key regulators in this field, the METTL family plays multiple roles in RNA methylation, PTMs of proteins, and metabolic regulatory networks by virtue of its unique methyltransferase activity [3]. From embryonic development to tumorigenesis, from neurodegenerative diseases to metabolic disorders, the dynamic modification behaviors of METTL family members profoundly affect life processes and pathological processes [4–6].

In recent years, studies have further revealed that the functions of METTL family members are far beyond the scope of a single “modifying enzyme”. For example, METTL16 not only catalyzes the m6A modification of U6 snRNA to regulate pre-mRNA splicing, but also maintains methylation metabolic homeostasis by stabilizing the mRNA of S-adenosylmethionine (SAM) synthetase, thus forming a “modification-metabolism” bi-directional regulatory loop. This multidimensional mode of action makes the METTL family a key hub connecting epigenetics, metabolic reprogramming and disease development [7]. With the breakthroughs in cryo-electron microscopy and single-cell sequencing, the three-dimensional structures and tissue-specific functions of METTL family members are becoming clearer. However, current research still faces multiple challenges: the molecular logic of substrate selectivity, the spatiotemporal regulation of dynamic modifications: (1) How are the METTL family members precisely regulated during the cell functions? (2) How METTLs enzyme activity affect nucleic acid modifications and protein PTMs? (3) How to achieve clinical translation of METTL inhibitors, due to their tissue-specific delivery and off-target effects are still a major obstacle.

This review systematically examines the structural features, molecular functions, and pathophysiological regulatory networks of METTLs, focusing on their diagnostic and therapeutic potential—particularly through METTL inhibitors. By evaluating mechanistic insights and clinical applicability, we critically assess their translational prospects as emerging therapeutic targets.

## Basic structure and functions of METTLs

The METTLs family of proteins is comprised of an N-terminal RNA-binding domain, a methyltransferase (MTases) domain (MTD), and a C-terminal vertebrate conserved region (VCR) conservation domain [8]. The first 40 or so amino acids at the N-terminus make up the RNA-binding structural domain, which contains several positively charged amino acid residues [9].

**Fig. 1:**
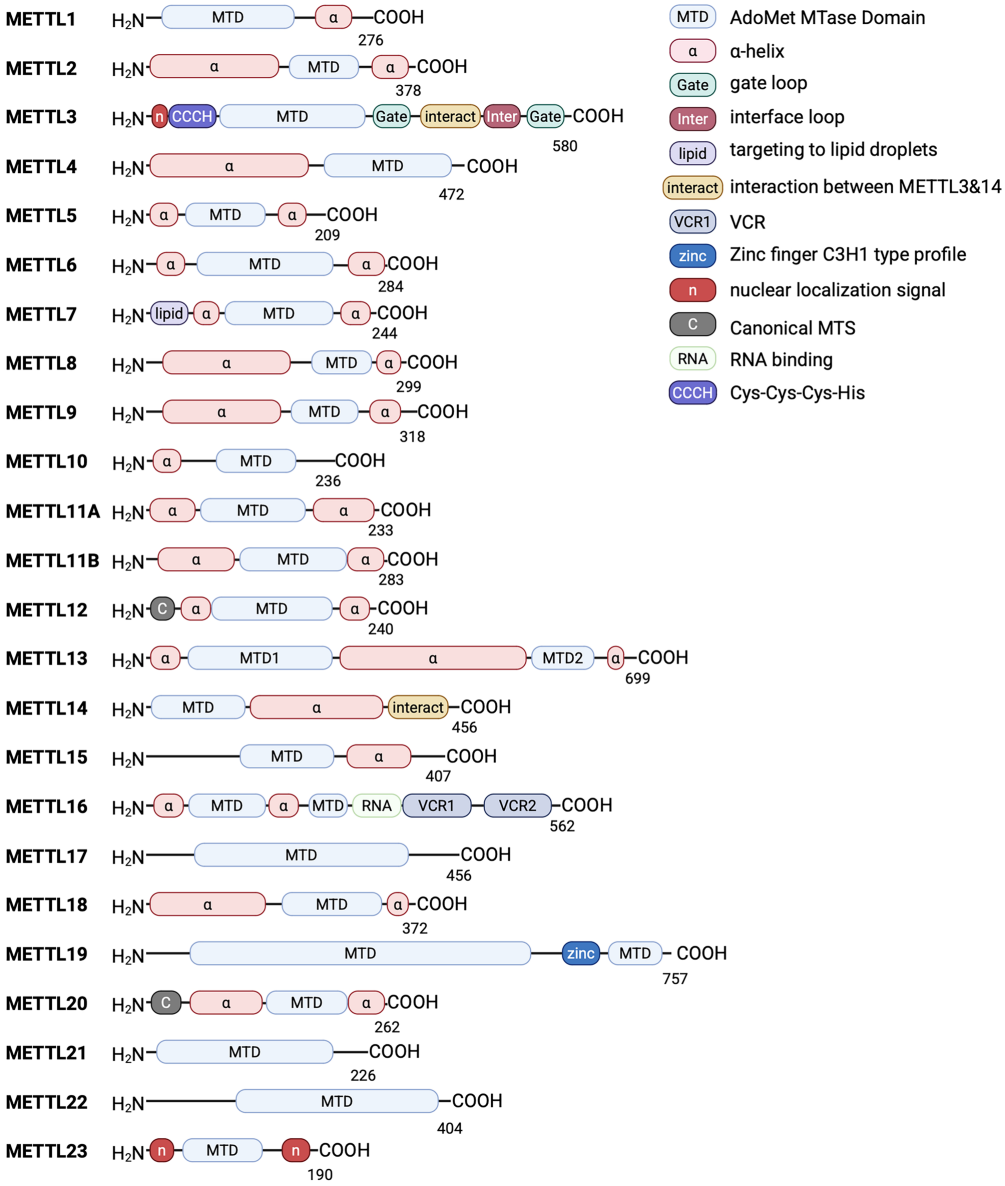
Domain architecture of human METTLs. The current study identified the basic structure of 23 METTLs. For all METTL proteins, residue numbers are based on human protein sequences. Each METTL protein contains one AdoMet MTase domain (except METTL16, which has two MTD) and 1–3 α-helical structures. Differences in substrate recognition function may be related to protein modules such as gate loop, Zinc finger, Cys-Cys-Cys-His. Other protein modules, such as the nuclear localization signal, RNA binding, target to lipid drops, function as localization aids

While most METTL family members exhibit a conserved SAM-binding domain, their specific structural features vary based on substrate specificity. This domain contains an ordered polypeptide loop, an NPPF motif, and a disordered loop [10]. The NPPF motif is the active site of MTD, which promotes the binding of METTL proteins to substrate RNA and induces RNA methylation [11]. Disordered loops can regulate the binding ability of METTL proteins to RNA through charge states. The function of the VCR structural domain, also known as the conserved region, has not yet been elucidated. Some studies suggest that the VCR structural domain may be involved in the regulation of RNA splicing and methylation [12]. To advances the comprehensive understanding of this biologically significant gene cluster in epigenetic regulation, we systematically delineate the structural architecture of the METTL protein family (Fig. 1).

### DNA/RNA epigenetic modification by METTLs

**Fig. 2:**
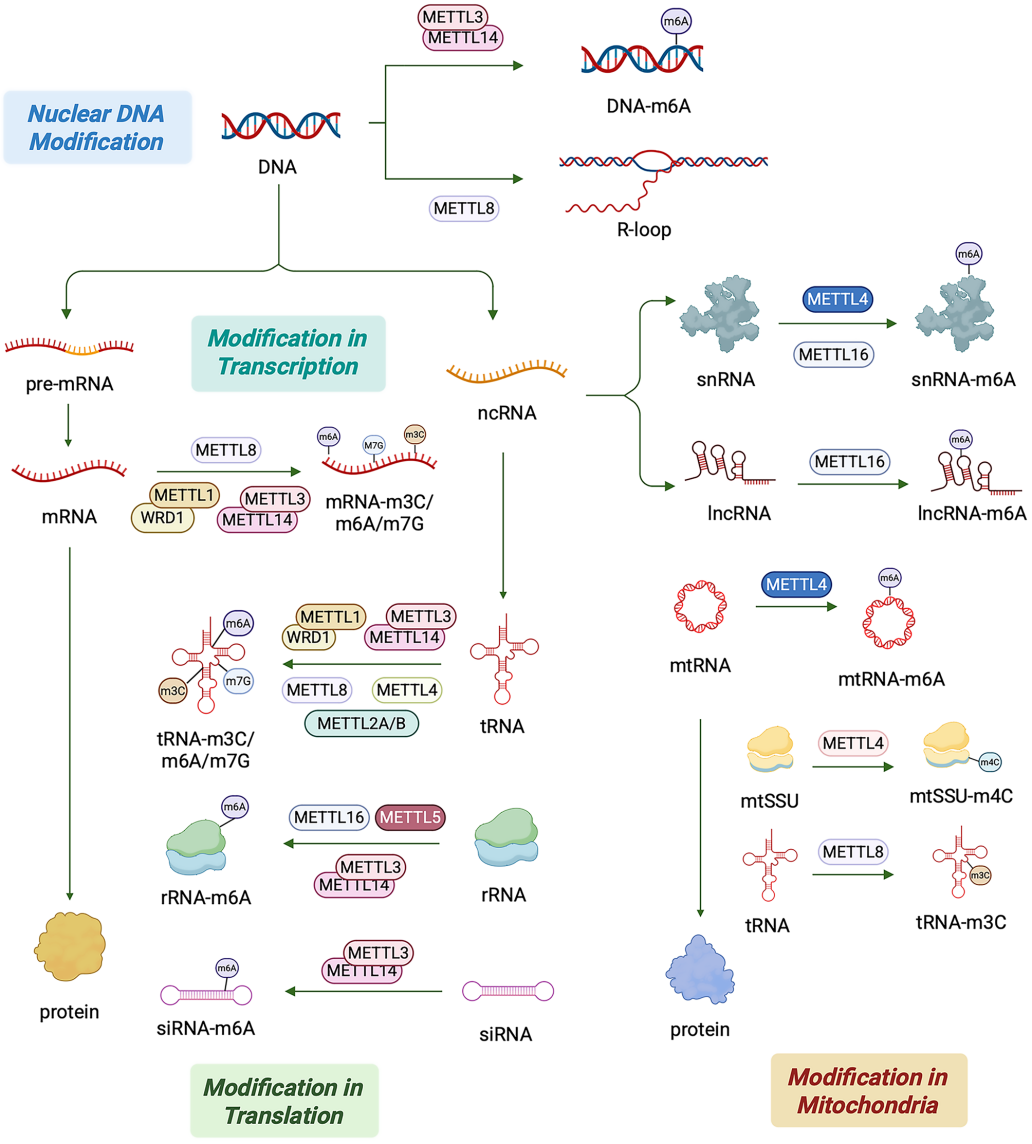
Schematic diagram of METTLs involved in epigenetic regulatory pathways. This figure summarizes the epitope modification functions of METTLs at different levels, including epitope modification of nuclear and mitochondrial DNA and their transcriptional and translational processes. METTLs can affect gene expression by regulating DNA structure, mRNA efficiency, and tRNA stability by means of epigenetic modifications such as m^6^A, m^3^C, and m^7^G. In addition, METTLs are capable of m^6^A modification of snRNA and lncRNA

Epigenetics refers to heritable changes in gene expression without altering the nucleotide sequence of the gene and includes DNA methylation, histone modifications and RNA modifications [13]. METTL family proteins have 11 family members that regulate transcript splicing and translation by participating in modifications such as m^7^G, m^6^A, 5-methylcytosine (m^5^C) and other modifications that affect related signaling pathways [1]. Various methylation modifications have a wide range of effects on RNA secondary structure folding, stability and function, affect physiological processes, and are closely related to many human diseases (Fig. 2).

#### M^7^G modifications

M^7^G represents a critical modification in various RNA species, including modification has been found in mRNA, tRNA and rRNA [14]. The first identified m^7^G reader is eIF4E, which recognizes the m^7^G cap structure to initiate mRNA translation [15]. Similar to eIF4E, the nuclear cap-binding protein (NCBP) complex can influence mRNA transcription, splicing, transport, translation, and degradation [16]. Additionally, the m^7^G within mRNA can be selectively recognized by the quaking protein (QKI) to regulate mRNA stability and translation under stress conditions [17].

It was determined that METTL1/WD repeat structural domain 4 (WDR4) complex are responsible for methyl transfer during the process of modification and are key regulators as m^7^G writing enzymes [18]. There is dynamic regulation of these modifications. When heat shock and oxidative stress occur, there is a significant increase in the abundance of m^7^G modifications in the mRNA CDSs and 3’UTR regions, and a significant decrease in the abundance of m^7^G modifications in the 5’UTRs, which ultimately leads to an increase in mRNA translation efficiency [19]. The m^7^G methylation of *Atf5* mRNA by METTL1 upregulates the expression of cell cycle-related proteins, which in turn affects cardiomyocyte proliferation [20]. Besides, METTL1 is similarly involved in the post-transcriptional regulation of neuronal gene expression and influences central neurogenesis [21].

#### M^3^C modifications

M^3^C is predominantly localized to the anticodon loop (position C32) of specific tRNAs, widely found in eukaryotic cytoplasmic and mitochondrial tRNAs. Current research suggests that m^3^C may affect the precise pairing between codons and anticodons [22]. Moreover, m^3^C is crucial for tRNA structure and folding, which is essential for translation fidelity [23].

METTL2A/B, METTL6 and METTL8 all primarily modify tRNAs in the cytoplasm, although the substrates and activation processes differ slightly. METTL2A and METTL2B both modify arginine, serine, and threonine tRNAs, and no functional differences have been detected except that the activity of METTL2B is only 1/10 of that of METTL2A, and the modification of METTL2A/B requires the involvement of a DALR anticodon binding domain containing 3 (DALRD3) protein and the t6A37 modification [24]. METTL6 modifies only serine tRNA. Seryl-tRNA synthetase (SerRS) enhances the methylation activity of METTL6 [22]. METTL8 is involved in m^3^C32 methylation modification of isoleucine in the cytoplasm and serine and threonine tRNAs in mitochondria, mRNA and long-stranded non-coding RNAs (lncRNA) [25]. SUMOylated METTL8 affects the organization of genes in or proximal to the nucleolus, promoting the formation of R-loops—three-stranded DNA: RNA hybrid structures that adversely affect genomic stability and may promote tumorigenesis [26].

#### M^6^A modifications

M^6^A serves dual roles in biological processes, functioning not only as an RNA regulatory modifier but also contributing to DNA demethylation, chromatin accessibility, and gene transcription [27]. M^6^A modification exerts its biological effects through multiple mechanisms. m^6^A can recruit specific binding proteins—known as m^6^A readers—to mediate downstream pathways. m^6^A is directly recognized by YTH domain proteins (YTHDF). YTHDF1 promotes interaction between m^6^A-modified mRNA and translation initiation factors, thereby facilitating translation initiation [28]. YTHDF2 is involved in the degradation process of mRNA, affecting its translational status and lifespan [29]. m6A modification can also directly alter the local structure of mRNA and lncRNA, promoting binding with heterogeneous nuclear ribonucleoprotein C (HNRNPC). The binding activity of HNRNPC influences the abundance and alternative splicing of target mRNAs [30].

Functionally, m6A-related proteins are categorized into writer, eraser and reader, and METTL3, METTL5, METTL14 and METTL16 all belong to writer. During the process of m^6^A modification, a variety of other proteins, collectively termed the m6A METTL-associated complex (MACOM), comprising HAKAI, VIRMA, ZC3H13 and RBM15. MACOM influences substrate binding, the efficiency of catalysis, stability and substrate selectivity [31].

The METTL3/METTL14 complex is by far the most widely studied m^6^A writers [32]. ‌METTL3 catalyzes m6A modifications in approximately 95% of cellular mRNAs and additionally targets diverse non-coding RNA species, including tRNAs, snRNAs, pre-miRNAs, and lncRNAs [33]. METTL3 also maintains cardiomyocyte remodeling as well as spermatogonial stem cell (SCC) homeostasis [34–37]. METTL14 alone has no MTase activity. However, METTL14 is involved in the regulation of histone modification in addition to forming a complex with METTL3 to modify various types of RNAs in the nucleus, cytoplasm and mitochondria [38]. METTL4 performs m^6^A modification of mRNA, snRNA, microRNAs and mitochondrial DNA (mtDNA) [39] which is involved in the regulation of glucose, lipid and testosterone metabolism [40, 41]. METTL5 specifically mediates methylation modification of mRNA and 18 S rRNA [42]. METTL16 in the nucleus mediates m6A modification of U6 snRNA and mRNA [43] and coordinates DNA repair in erythroid progenitor cells and enhances synaptic plasticity associated with memory and learning processing [7, 44]. In addition, METTL3, METTL5 and METTL16 are all involved in the differentiation of mESCs and skeletal muscle tissues [45–47].

#### M^4^C modifications

Compared to other means of RNA epimodification, N4-methylcytosine (m^4^C) has been much less studied. Recently m^4^C has been shown to be the major methylated base of eukaryotic cytoplasmic and mitochondrial rRNAs [48, 49]. METTL15 methylates mitochondrial 28 S mitochondrial small subunits (mt-SSU, 12 S) [50] which assembly intermediates can be divided into “early” and “late” stages. METTL15 is involved in the late stage of mtSSU assembly and promotes its maturation [51]. Interestingly, a study showed that mitochondria lacking METTL15-mediated modification of m^4^c were able to maintain reasonable translational efficiency despite the fact that METTL15 is essential for mitochondria [52]. Whether the METTL15-mediated modification of m^4^C is a functional redundancy or whether there are other mechanisms in balance with it then remains to be further investigated.

### Protein epigenetic modification by METTLs

PTMs are covalent modifications through protein-protein interactions that control the activation and inactivation of cellular pathways [53]. Fourteen of the METTL family interact with proteins, most with lysine as a substrate and a few with histidine or arginine.

#### Lysine methylation

Lysine methylation is a well-defined, reversible modification observed in histone and non-histone targets. Mammalian hepta-β chain (7BS) lysine (K)-specific MTase (KMT) primarily targets the translational apparatus, methylating eukaryotic translation elongation factor 1α (eEF1A) and eukaryotic translation elongation factor 2 (eEF2) [54]. In addition to eEF1A, METTLs KMT targets a variety of other substrates. METTL10 (eEF1A-KMT2), METTL13 (FEAT, eEF1A-KNMT) and METTL21B (eEF1A-KMT3) were all identified as using eEF1A as a substrate [55, 56]. METTL21B can introduce mono-, dimethyl- and trimethylation. However, the physiological significance of such dynamic methylation changes is unclear [57, 58]. METTL12 (CS-KMT) mediates trimethylation of citrate synthase (CS), thereby inhibiting the catalytic activity of CS [59]. METTL20 (ETFβ-KMT) introduces mono-, dimethyl- and trimethylation on the β-subunit of the electron transfer flavoprotein (ETF) and is involved in the regulation of dehydrogenases [60]. METTL21A (HSPA-KMT) was shown to trimethylate the ‘lid’ structural domain of Hsp70 [61]. Methylation modification of Heat Shock Protein Family A (HSPA) by METTL21A may affect angiogenesis [62]. METTL21D (VCP-KMT) can trimethylate valine-containing protein (VCP), thereby reducing its ATPase activity [63]. Whether the substrate of METTL21C is VCP, HSPA, or 5-alanyl-tRNA synthetase 1 (AARS1) requires further exploration [64].

#### Arginine methylation

The presence of multiple nitrogen atoms in arginine (Arg) makes its methylation more complex than that of lysine. Currently, it is believed that the “reader” proteins that catalyze arginine methylation are mainly the subfamily of arginine methyltransferases (PRMT), which also belong to the 7BS MTases. However, recent studies have revealed that METTL23 can also catalyze arginine methylation [65]. METTL23 has been found to interact with the transcription factor subunit GABPA to regulate thrombopoietin and ATP5B. This pathway may also be involved in human cognitive function [66]. While METTL23 remains the only METTL family member currently known to possess intrinsic arginine methyltransferase activity, the extensive functional diversity observed within this enzyme family suggests that future research may reveal additional METTLs capable of arginine methylation. The structural plasticity of the catalytic domains in METTL enzymes, combined with their capacity to recognize diverse substrates, leaves open the possibility that underappreciated arginine methyltransferase activities exist among other METTL members. However, no such activities have been experimentally validated to date beyond METTL23.

#### Histidine methylation

More than 13% of protein methylation events in the human methylome are attributed to modifications of protein histidine residues [67]. Histidine may be methylated on the nitrogen at the 1 (Nπ) or 3 (Nτ) position of the imidazole ring, yielding the isomers Nπ-methylhistidine (1-methylhistidine) or Nτ-methylhistidine (3-methylhistidine), respectively. METTL9 could methylate His-x-His (HxH)-containing proteins, including the immunomodulatory protein S100A9 and the NDUFB3 subunit of mitochondrial respiratory complex I [68]. METTL9-catalyzed S100A9 histidine methylation inhibits the anti-Staphylococcus aureus activity of neutrophils [69]. METTL18 mediates τ-N-methylation on His245 of ribosomal protein large subunit (RPL) 3 and regulates translational elongation to maintain protein homeostasis [70]. However, the biological significance of protein histidine methylation remains largely unclear [71].

#### Free amino acid methylation

N-terminal methyltransferase is a PTM that affects many biological processes by regulating protein stability, protein-DNA interactions and protein-protein interactions. N-terminal methylation primarily occurs after methionine cleavage. N-terminal methylation is thought to be an irreversible process, and no validated N-terminal demethylases have been identified [72]. New studies have shown that METTL11A mono-, di- and trimethylates the N-terminal X-Pro-Lys (X-P-K) common sequence. METTL11A substrates that have been validated include the tumor suppressor retinoblastoma protein (RB), the oncoprotein SET, the transcription factor Kelch-like protein 31, and many myosin light chains and ribosomal proteins [73]. The current study suggests that METTL11A is involved in the differentiation process of myofibroblasts and aging [74, 75]. METTL11B also recognizes and methylates XPK common sequences. METTL11A and METTL11B have similar localization and expression patterns but differ in catalytic activity, with METTL11B being primarily a monomethylase [76].

### Other epigenetic modification

Some other METTLs do not directly use genetic material or proteins as substrates, but recognise some small molecular moieties that have an indirect effect. METTL7A and its homologue METTL7B have a membrane-associated thiol MTase (TMT) activity. TMT1A does not methylate endogenous thiols, including cysteine and glutathione or other small molecules with specificity for N and O-methyltransferases. METTL7A selectively methylates exogenous thiol-containing substrates including 7α-thiopyrrolactone, dithiothreitol, 4-chlorothiophenol, and mephedrone [77]. METTL7A and METTL7B achieve resistance to histone deacetylase (HDAC) is through methylation and inactivation of zinc-binding thiols [78]. METTL7A may be involved in the regulation of cerebellar neural circuits, the embryonic developmental cycle and osteogenic differentiation [79–81]. METTL17, on the other hand, recognizes iron-sulfur (Fe/S) clusters in mitochondria and regulates the regulation of mitochondrial protein expression [82]. It has been suggested that METTL17 may be involved in the early stages of mtSSU assembly and act as a platform for METTL15 recruitment [83]. In addition, the METTL family plays a central role in RNA epigenetics, protein function regulation and metabolic reprogramming through precise substrate recognition and dynamic modification networks. Future studies need to combine single-cell multi-omics with artificial intelligence to resolve tissue-specific modification profiles and develop tissue-selective inhibitors to overcome the off-target effects of existing therapies.

### Cooperative network among METTL members

**Fig. 3:**
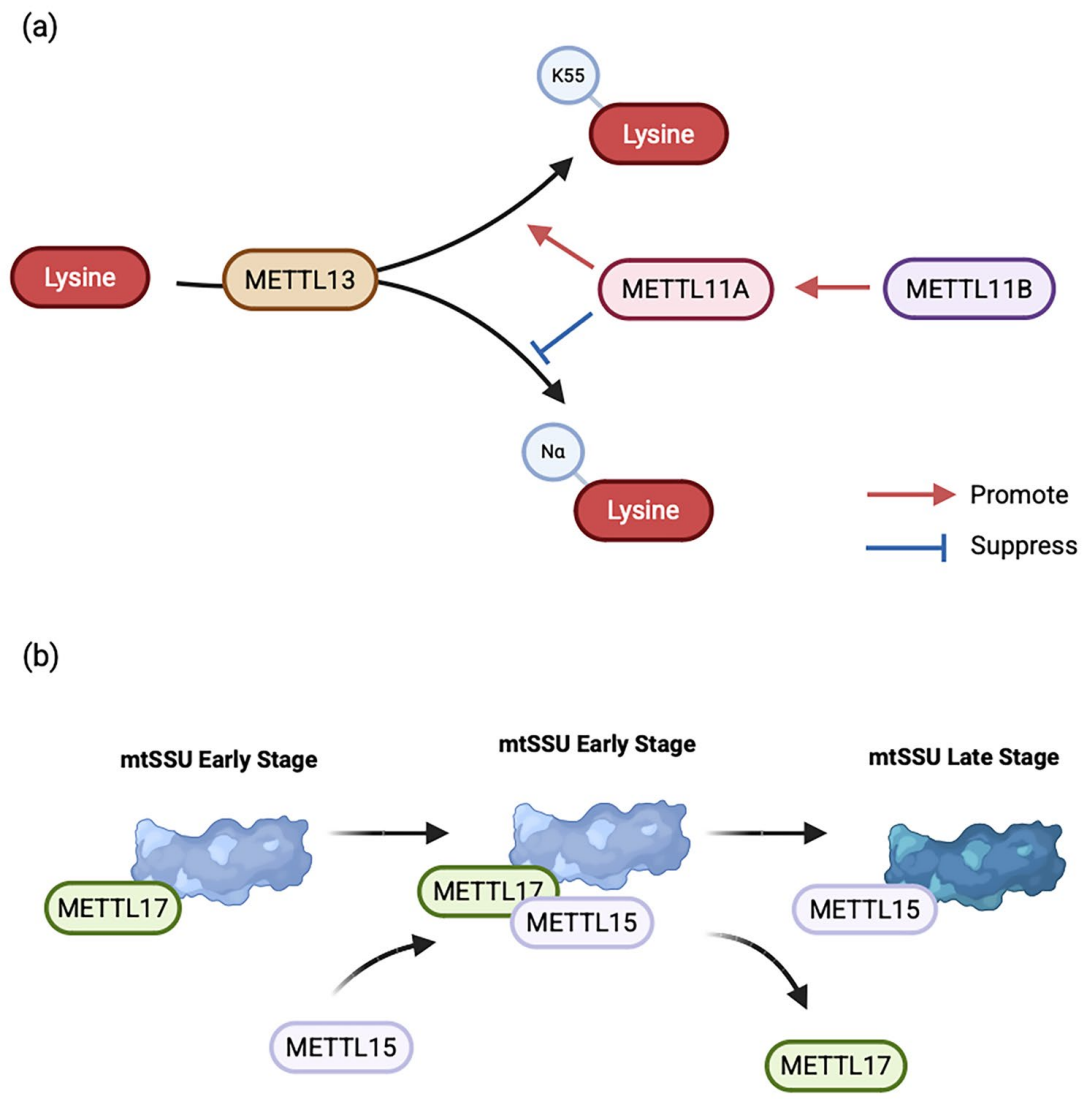
Diagram of the collaborative network among METTL members. Interactions also occur between METTL members. (**a**) METTL11B can promote the activity of METTL12. Meanwhile, the methylation of METTL13 by METTL12 is subject to dual regulation. (**b**) During mtSSU assembly, METTL17 serves as the recruitment platform for METTL15

Proteins within the METTL family not only directly modify substrate methylation but also exhibit interactions among themselves. In addition to METTL3 and METTL14 forming complexes, METTL11A and METTL11B can also bind to form heterotrimers. METTL11B may enhance METTL11A’s trimethylating enzyme activity by increasing METTL11A’s stability and affinity for substrates [84]. Research indicates that METTL13 inhibits the activity of METTL11A. Concurrently, METTL11A promotes the K55 methylation activity of METTL13 while suppressing its Nα-methylation activity. When METTL11A, METTL11B, and METTL13 coexist, the regulatory effect of METTL13 takes precedence over that of METTL11B [85]. Additionally, a study on the assembly process of mtSSUs revealed an interaction between METTL15 and METTL17. METTL17 serves as a platform for recruiting METTL15. Subsequent release of METTL17 enables a conformational change in METTL15 to recognize its substrate [83]. This suggests that despite sharing similar reaction substrates, they do not represent functional redundancy (Fig. 3).

## Physiological and pathological roles of METTLs

**Fig. 4:**
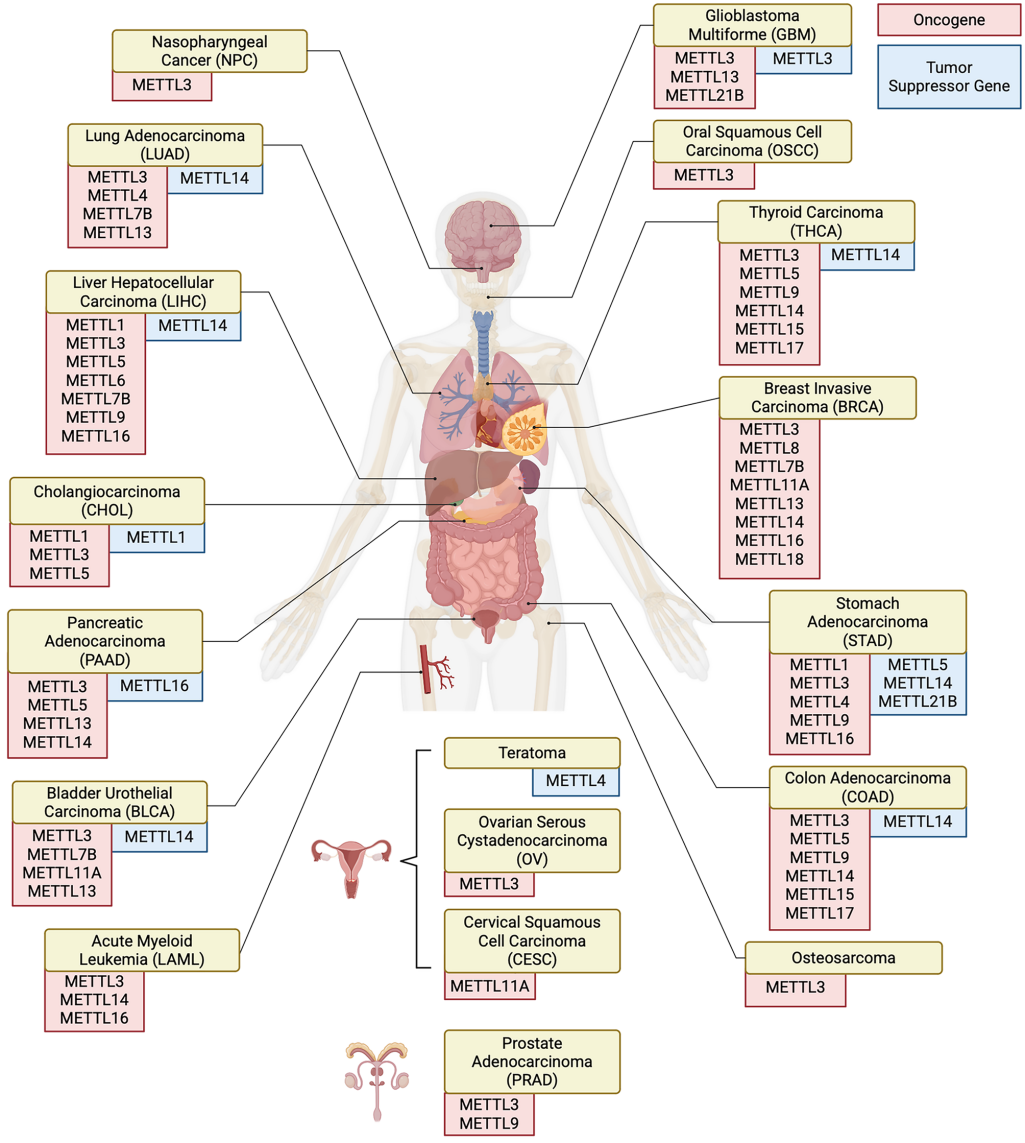
Summary of the role of METTL in tumors. METTL proteins act as oncogenes or tumor suppressor gene in the development of different tumors. Red, suggested as a promoting role. Blue, suggested as a suppressing role. The majority of the METTLs have been observed to promote tumorigenesis. METTL1, METTL3, METTL5, METTL14, METTL16, and METTL21B have been shown to act as oncostatic agents in certain tumors. It has been demonstrated that METTL1, METTL3 and METTL14 play a dual regulatory role in CHOL, GBM and STAD respectively

The METTL family, as the core executors of epigenetic regulation, profoundly affects the homeostatic balance of life activities and disease processes through a dynamic network of methylation modifications. At the physiological level, METTL members regulate cell fate and tissue development through precise substrate modifications. At the pathological level, dysfunction of the METTL family is widely involved in disease genesis. Notably, METTL members often exhibit “double-edged sword” properties in disease, and the high spatiotemporal specificity of their regulatory networks poses a challenge for targeted therapy. For example, METTL3 promotes tumorigenesis in colorectal cancer by enhancing translation of oncogenic mRNAs, yet it suppresses growth in ovarian cancer by regulating IL-1β secretion—highlighting how regulates host immune response determines functional outcomes [86]. METTL14, commonly recognized as a partner of METTL3, also demonstrates context-dependent functions. While it frequently enhances METTL3’s oncogenic activity, certain studies suggest opposing roles in liver and metabolic diseases—a phenomenon potentially attributed to differences in RNA substrate specificity or tissue-specific expression patterns. Emerging evidence suggests that multiple METTL family members exhibit context-dependent functional outcomes, with studies reporting contradictory roles across different biological contexts. This is due to insufficient exploration of the underlying mechanisms and limited comparative evidence being available. These areas warrant further investigation through more qualitative and quantitative research to identify key factors regulating METTL. Overall, while descriptive associations between METTLs and disease phenotypes are informative, careful comparison, evaluation of conflicting evidence, and weighting of the evidence base are essential for a balanced understanding of this protein family (Fig. 4).

### METTLs in tumor-related mechanisms

METTLs play pivotal roles in tumorigenesis across diverse biological systems, with their functions varying significantly. In this review, we systematically summarize their involvement in key signaling pathways, including AKT, cell cycle, MYC, ferroptosis and tumor microenvironment.

#### AKT signal pathway

The AKT represents a critical molecule within kinase signaling networks and has been implicated in numerous cancer-related cellular processes. Dysregulation of METTL family members influences the AKT pathway in oncogenic contexts. METTL1 promotes the expression of EGFR through m^7^G modification, which in turn promotes the phosphorylation of AKT, which in turn contributes to tumorigenesis [87]. The METTL3/METTL14 complex can activate the AKT pathway by promoting the expression of ADAMTS9 and miR-380-3p to promote tumor angiogenesis and EMT [88, 89]. Additionally, METTL3 inhibits PTEN, counteracting its suppressive effect on the AKT pathway [90]. However, there are also two independent studies that point to a possible inhibitory effect of METTL3/METTL14 on the EGFR/PI3K/AKT pathway [91, 92]. Alternatively, METTL13 is able to activate AKT downstream by promoting protein kinase PAK2 or PI3K pathways [93].

#### Cyclins

**Fig. 5:**
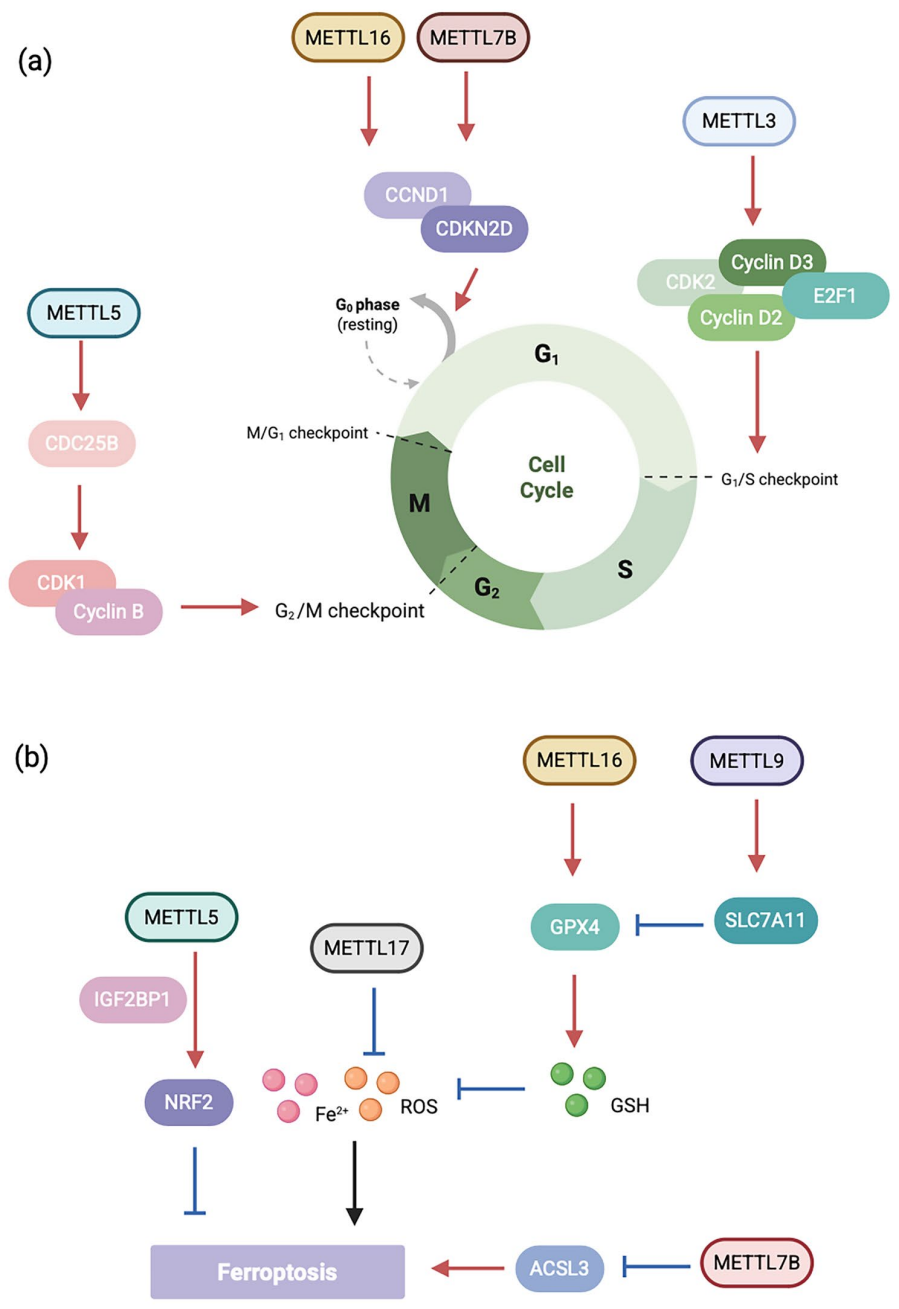
Regulation of the cell cycle and ferroptosis by the METTL family. METTL family members are involved in cancer development by mediating aberrant cell signaling pathways. The blue and red arrows symbolize the process of downstream inhibition and promotion by METTLs, respectively. (**a**) METTLs exert their effects by regulating cell cycle-associated proteins. (**b**) METTLs regulate ferroptosis by influencing reactive oxygen species and associated proteins

Cell cycle regulation is closely related to the malignant progression of tumors. METTL3 enhances CDC25B expression which activates CDK1/cyclin B to initiate G2/M transition via m6A, fueling HNSCC tumorigenesis [94]. METTL3 knockdown significantly downregulated the expression of cell cycle-associated proteins (hosphor-CDC2, CDK2, cyclin D2, cyclin D3, E2F1, and hosphor-Rb), resulting in G1 phase arrest of the cell cycle and inhibiting uveal melanoma cell proliferation and colony formation [95].Compared to normal tissues, METTL7B expression is elevated in the majority of Non‑Small Cell Lung Cancer (NSCLC) cases. Silencing METTL7B leads to downregulation of the cell cycle regulator cyclin D1 (CCND1) and upregulation of cyclin-dependent kinase 4 inhibitor (CDKN4), resulting in G0/G1 phase arrest in cancer cells and a significant reduction in their proliferative capacity both in vitro and in vivo [96]. METTL16 is highly expressed in gastric cancer patients. METTL16 enhances the stability of CCND1 mRNA through methylation modification, thereby increasing CCND1 expression and promoting the proliferation of gastric cancer cells [97]. We mapped the mechanism of action of METTL in the cell cycle (Fig. 5).

#### METTL in ferroptosis

Cancer cells have high iron content and correspondingly higher sensitivity to ferroptosis. METTL16 enhances GPX4 expression via m6A modification, thereby inhibiting ferroptosis and promoting breast cancer progression [98]. METTL9 knockdown reduces the expression levels of SLC7A11, a key inhibitor of ferroptosis, thereby indirectly inducing ferroptosis in hepatocellular carcinoma (HCC) [99]. Inhibition of METTL17 disrupts mitochondrial function and energy metabolism, elevates intracellular and mitochondrial lipid peroxidation and ROS levels, thereby increasing Colorectal Cancer (CRC) cell susceptibility to ferroptosis [100]. Additionally, METTL17 may link mitochondrial translation to lipid peroxidation-mediated ferroptosis. METTL7B expression promotes m6A modification on Acyl-CoA Synthetase Long Chain Family Member 3 (ACSL3) mRNA, thereby reducing ACSL3 gene expression and ultimately inhibiting ferroptosis in bladder cancer [101]. Nuclear factor erythroid 2-related factor 2 (NRF2) has been implicated in ferroptosis across multiple tumor types. METTL5-mediated m6A modification enhances NRF2 mRNA stability via the reader protein IGF2BP1, thereby inhibiting ferroptosis in gastric carcinoma [102].

#### MYC signal pathway

MYC oncoproteins are involved in multiple biological functions in cancer. Current studies have shown that MYC has an effect on tumor immune response, metabolism, cell cycle, apoptosis, autophagy, pyroptosis, metastasis and angiogenesis. METTL5 promotes the translation of MYC and increases the stability of MYC mRNA, facilitating the expression of MYC, which in turn activates the expression of glycolytic genes such as lactate dehydrogenase A (LDHA) and ultimately serves to promote tumorigenesis and metastasis [103–105]. METTL3 targets the 3′ UTR (adjacent to the stop codon) of the c-Myc transcript to install m6A modifications, thereby promoting c-Myc stability via the reader protein YTHDF1 in oral squamous cell carcinoma (OSCC) cohort [106].

#### METTL in tumor microenvironment

Tumor-associated immunity has emerged as a prominent research focus in recent years. The irreversible dysfunctional state of T cells within the tumor microenvironment (TME) results in many patients not responding effectively to immunotherapy. The METTL3/METTL14 complex inhibits CD8 + T immune cell dysfunction and differentiation, indirectly promoting the ability of CD8 + T cells to eliminate tumors [107, 108]. METTL1 inhibits the function of CD8 + T immune cells and promotes tumor-associated macrophages (TAM) infiltration [109]. METTL21B was found to promote macrophage polarization from M1 to M2, modulate immune checkpoint expression and promote tumor immune evasion in low-grade gliomas [110]. METTL5 promotes anti-tumor immune function and inhibits tumor-associated immune escape in PBMCs cells [102].

**Fig. 6:**
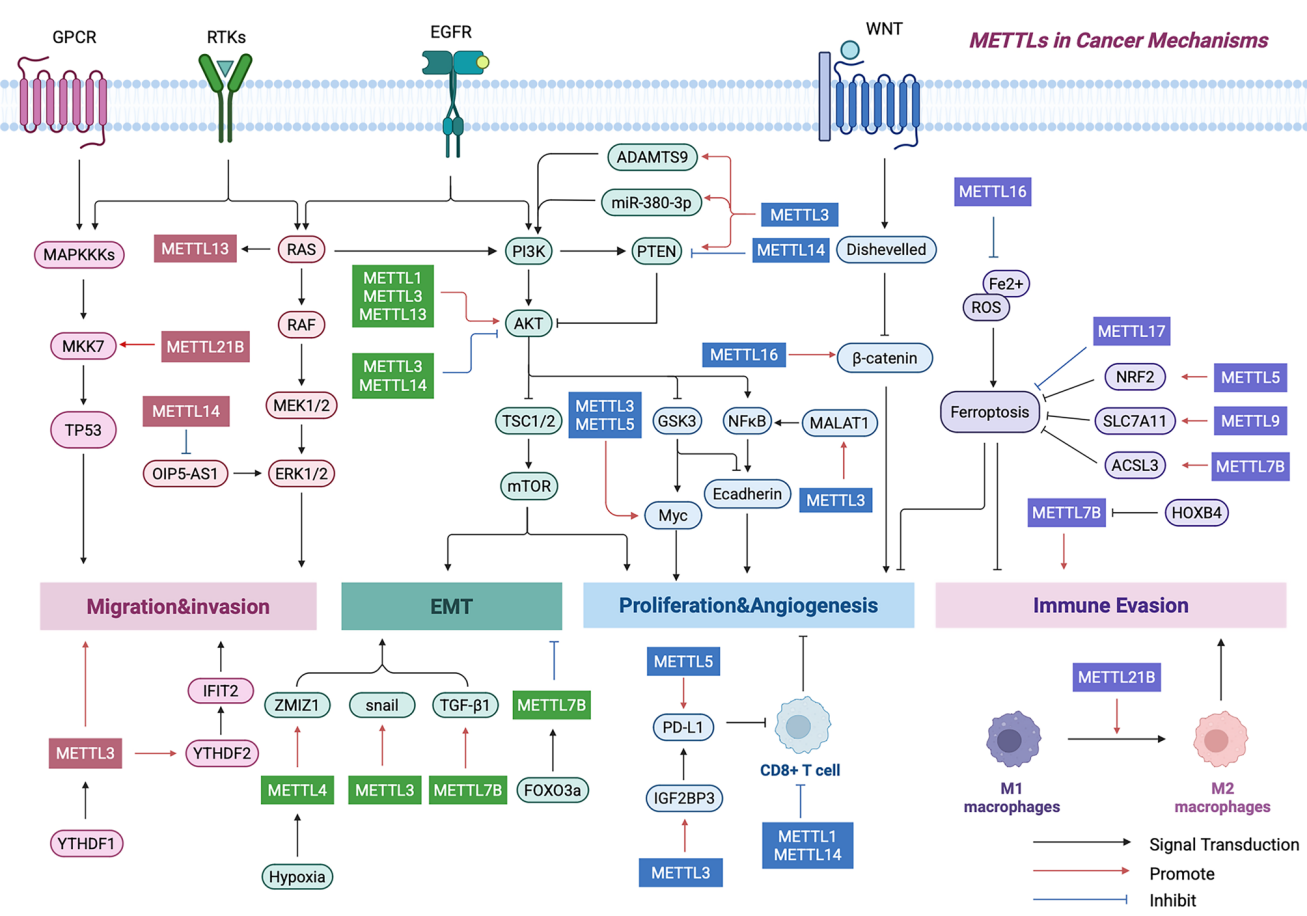
Mechanism of METTL family proteins in cancer development. METTL family members are involved in cancer development by mediating aberrant cell signaling pathways. The black arrows thus represent the conduction pathways of the signalling pathways. The blue and red arrows symbolize the process of downstream inhibition and promotion by METTLs, respectively. (**a**) METTL14 and METTL21B can promote tumor malignant invasion via the ERK1/2 pathway and the MKK7 pathway. METTL3, on the other hand, promotes metastasis by interacting with the READER protein YTHDF1/2. (**b**) METTL1, METTL3 and METTL13 can activate the PI3K/AKT pathway in different ways to activate the downstream mTOR, myc and NFκB pathways to promote tumorigenesis, angiogenesis and EMT. whereas METTL14 can inhibit the PI3K/AKT pathway to play the opposite role. In addition, METTL1, METTL3, METTL5 and METTL14 can inhibit the normal tumor killing function of CD8 + T cells, resulting in tumor progression. (**c**) Induction of ferroptosis is a new idea for tumor therapy. METTL5, METTL7B, and METTL9 can inhibit tumor progression and promote immune infiltration by facilitating the onset of ferroptosis. Up-regulation of METTL16 and METTL17 inhibits the process of ferroptosis. In addition, METTL21B was able to promote tumor immune escape by inducing polarization of M1 macrophages to M2 macrophages

Overall, most of the METTL proteins play a pro-oncogenic role in tumors. In addition to the well-studied mechanistic pathways mentioned above, they can also contribute to tumorigenesis through the regulation of specific ‘reader’ proteins YTHDF, amino acid metabolism, the RAS, and histone deacetylase (HDAC) pathway [111–113]. A few METTLs, such as METTL1, METTL14 and METTL16, also showed oncogenic effects, to systematically delineate the distinct roles of METTL family members across tumor types, we generated a comprehensive schematic (Fig. 6).

Additionally, the roles between METTLs-mediated epigenetic modifications and tumorigenesis are complex and diverse, involving many regulatory pathways, various regulatory means and different regulatory effects. Further studies are needed to clarify the interactions of the regulatory network of METTLs and the hierarchical structure of the downstream pathway.

### METTLs in metabolic diseases

#### Diabetes mellitus

There is growing evidence that METTL3 is strongly associated with diabetes mellitus (DM), including insulin deficiency, hyperglycemia and insulin resistance. In β-cells, METTL3 regulates type 1 diabetes-associated innate immunity via redox modulation [126], and its deficiency disrupts insulin secretion [127]. METTL3 expression is increased in patients with type 2 diabetes (T2D) [114]. METTL3 reduces insulin sensitivity and is involved in glucose metabolism by increasing m6A methylation for fatty acid metabolism, hepatic gluconeogenesis [115]. In contrast, other studies have implied a protective effect of METTL3 against DM. METTL3-driven m6A modification in β-cells enhances insulin secretion, counteracting hyperglycemia and β-cell failure [116]. METTL3 also attenuates insulin secretion impairment in pancreatic β-cells by regulating MafA expression [117]. Beyond METTL3, METTL14 has also been implicated in T2D. Certain inhibitors have demonstrated promising efficacy in translational studies. For example, Dagliflozin inhibits high glucose-induced proliferation, oxidative stress and fibrosis by reducing Mettl3-induced m6A modification in Marcks mRNA [118] Semaglutide inhibits β-cell function through METTL14 signaling and regulation of the gut microbiota in T2D mice [119].

#### Obesity

METTL3 has been reported to play a key role in the development of obesity [120] which can drive lipid accumulation in a variety of cells by affecting lipid synthesis and catabolism [121]. Upregulation of METTL3 in myeloid cells also contributes to obesity by reducing the stability of DNA damage-inducible transcription 4 mRNA. Intermittent fasting to inhibit METTL3 activity and reduce m6A methylation levels attenuates apoptosis and lipid deposition and improves cardiac structure and function in obesity-associated cardiomyopathy [122]. However, other studies have shown a negative correlation between METTL3 expression and adipogenesis [123]. METTL3 was shown to upregulate the expression of cell cycle protein A2, a key regulator of mitotic clonal expansion, thereby promoting cell cycle transition and actively controlling adipogenesis [124]. In addition, deletion of METTL14 in the embryonic hypothalamus leads to defective neurogenesis in the arcuate nucleus of the hypothalamus, reduced production of feeding-related neurons, dysregulation of neurogenesis-related m^6^A-tagged transcripts, and ultimately obesity [125].

#### Non-alcoholic fatty liver disease

METTL3 is a key regulator of liver function and homeostasis, and increasing evidence suggests that METTL3 is critical for the development and progression of non-alcoholic fatty liver disease (NAFLD). In free fatty acid (FFA)-treated hepatocytes and NAFLD, m6A modification is increased, and aberrant m6A modification has been associated with lipotoxicity-induced increases in METTL3 [126]. METTL3 plays a dual role in NAFLD pathogenesis: (1) Promotes disease progression via MYC mRNA modification mechanisms [127]; (2) Exerts protective effects against non-alcoholic steatohepatitis by suppressing CCL2-mediated inflammation and CD36-dependent hepatic FFA uptake [128]. Concurrently, METTL16 emerges as a key driver of NAFLD progression in high-fat diet mouse models [129].

#### Osteoporosis

Osteoporosis (OP) is a prevalent global disease characterized by systemic disturbances in bone metabolism, leading to reduced bone density and changes in bone structure that greatly increase susceptibility to fracture due to brittleness. Imbalanced bone remodeling leads to OP. Although current drugs enhance the involvement of osteoblasts in bone formation, the underlying pathways remain unclear. METTL14 mediates Glut3 m^6^A methylation to improve OP under oxidative stress conditions [130]. Overexpression of METTL3 alleviates estrogen deficiency-induced OP [131]. Elevated METTL3 significantly promoted osteogenic differentiation of BMSCs and alleviated OP through the LINC00657/miR-144-3p/BMPR1B axis [132].

### METTLs in cardiovascular diseases

#### Ischaemic heart disease

METTL3 expression is significantly elevated in ischaemic cardiac tissues, and inhibition of METTL3 ameliorates myocardial fibrosis induced by myocardial infarction (MI) [133]. METTL3 increases miR-143-3p expression to inhibit protein kinase C ε (PRKCE) transcription to exacerbate cardiomyopathy (CM) firing in MI/reperfusion (MI/R) injury [80]. METTL3 was also able to inhibit activation of the tetanus kinase 2 (JAK2)/signal transducer and activator of transcription 3 (STAT3) pathway by up-regulating the expression of early growth response protein 1 (EGR1), thereby inhibiting mitosis, disrupting mitochondrial dynamics and ultimately exacerbating MI/R injury [134]. In turn, infarct size was reduced when METTL3 was silenced. Cardiac regeneration and cardiac function could be improved after MI via the METTL3- miR-143-Yap/Ctnnd1 axis [135]. Aerobic exercise training attenuates ischemia-reperfusion injury in mice by reducing the methylation level of METTL3-related m^6^A RNA in cardiomyocytes [136].

#### Cardiac hypertrophy and heart failure

METTL3 may be a deleterious factor in the development of cardiac hypertrophy. Emerging evidence suggests that overexpression of METTL3 controls left ventricular shortening fraction and ejection fraction, upregulates the expression of hypertrophic markers, induces myocardial fibrosis, impairs cardiac function and reverses the anti-hypertrophic effects of hawthorn acid, ultimately leading to pathological cardiac hypertrophy [137]. METTL3-mediated m6A modification of OTU Deubiquitinase 1 (OTUD1) exacerbates pressure overload-induced cardiac hypertrophy by deubiquitinating PGAM5 [138]. Increased METTL3 expression regulates mitogen-activated protein kinase (MAPK) and intracellular signaling pathways and further mediates CM hypertrophy, whereas silencing of METTL3 prevents the ability of CM to hypertrophy [139] In heart failure (HF), m6A methylation and METTL3 expression are elevated. Inhibition of METTL4 alleviates HF by regulating excess m6A in mitochondrial DNA [140]. METTL7B-induced histone lactylation prevents HF by improving cardiac remodeling [141]. Also, loss of function of METTL5 promotes pressure overload-induced cardiomyocyte hypertrophy and adverse remodeling [142].

#### Arrhythmia

METTL3 plays an important role in mediating the inflammatory response. M^6^A modification of METTL3 induces an increase in the expression of toll-like receptor 4 or TNF receptor-related factor 6, which activates the Nuclear Factor-κB (NFκB) signalling pathway. This process ultimately induces excessive sympathetic remodelling, increasing the incidence of VAs after MI and aggravating cardiac function [143]. Overexpression of METTL4 amplified cardiomyocyte mtDNA m^6^A results in spontaneous mitochondrial dysfunction and an atrial fibrillation phenotype [144].

#### Other cardiovascular diseases

The arteriovenous malformation phenotype is associated with low expression of METTL3, which subsequently affects angiogenesis in endothelial cells [145]. METTL3 plays a key role in vascular diseases including atherosclerosis (AS) [146], hypoxic pulmonary hypertension [147] and aortic dissection [148]. METTL4-mediated modification of mitochondrial DNA m6A coordinates the expression of genes related to mitochondrial function in macrophages, thereby promoting AS [149].

### METTLs in neurological diseases

METTL1 deficiency leads to reduced levels of m^7^G modification on Sptbn2 mRNA, which inhibits stability and translation, leading to impaired hippocampal neurogenesis and spatial memory in adult mice, and ultimately to Alzheimer’s disease (AD) [150]. METTL3 overexpression-mediated upregulation of RNA m6A rescues memory deficits and cognitive impairment in AD [151]. Rubinstein-Taibbi syndrome in which METTL1 is involved, may be associated with post-transcriptional modifications needed for rRNA maturation related. Correlative studies have shown that METTL17 acts as a Fe-S cluster checkpoint: translation of Fe-S cluster-rich OXPHOS proteins is promoted only when Fe-S cofactors are replete [152]. METTL3 attenuates hippocampal neuronal apoptosis and suppresses autism-like symptoms by regulating MALAT1/secretory frizzled-related protein 2/Wnt/β-catenin signaling [153]. In addition, some clinical reports have associated the METTL23 mutation with intellectual disability, accompanied by developmental delays and various dysmorphic features [154].

### Others

M^7^G modification mediated by METTL1 upregulates mt-tRF3b-LeuTAA expression, which exacerbates chondrocyte degeneration. leading to osteoarthritic Osteoarthritis (OA) [155]. METTL3 knockdown enhances cell invasion and migration in a METTL3/m^6^A/miR126 axis-dependent manner, thereby promoting the development of endometriosis [156]. A serum proteomic profile suggests that METTL18 may be associated with dysregulation of Fibromyalgia syndrome-associated proteins [157].Targeting METTL7A may provide a new strategy for the treatment of bisphosphonate-associated osteonecrosis of the jaw [158].

## Potential of METTLs in diagnosis and therapy

### Alteration of METTLs in disease prognostic roles

**Fig. 7:**
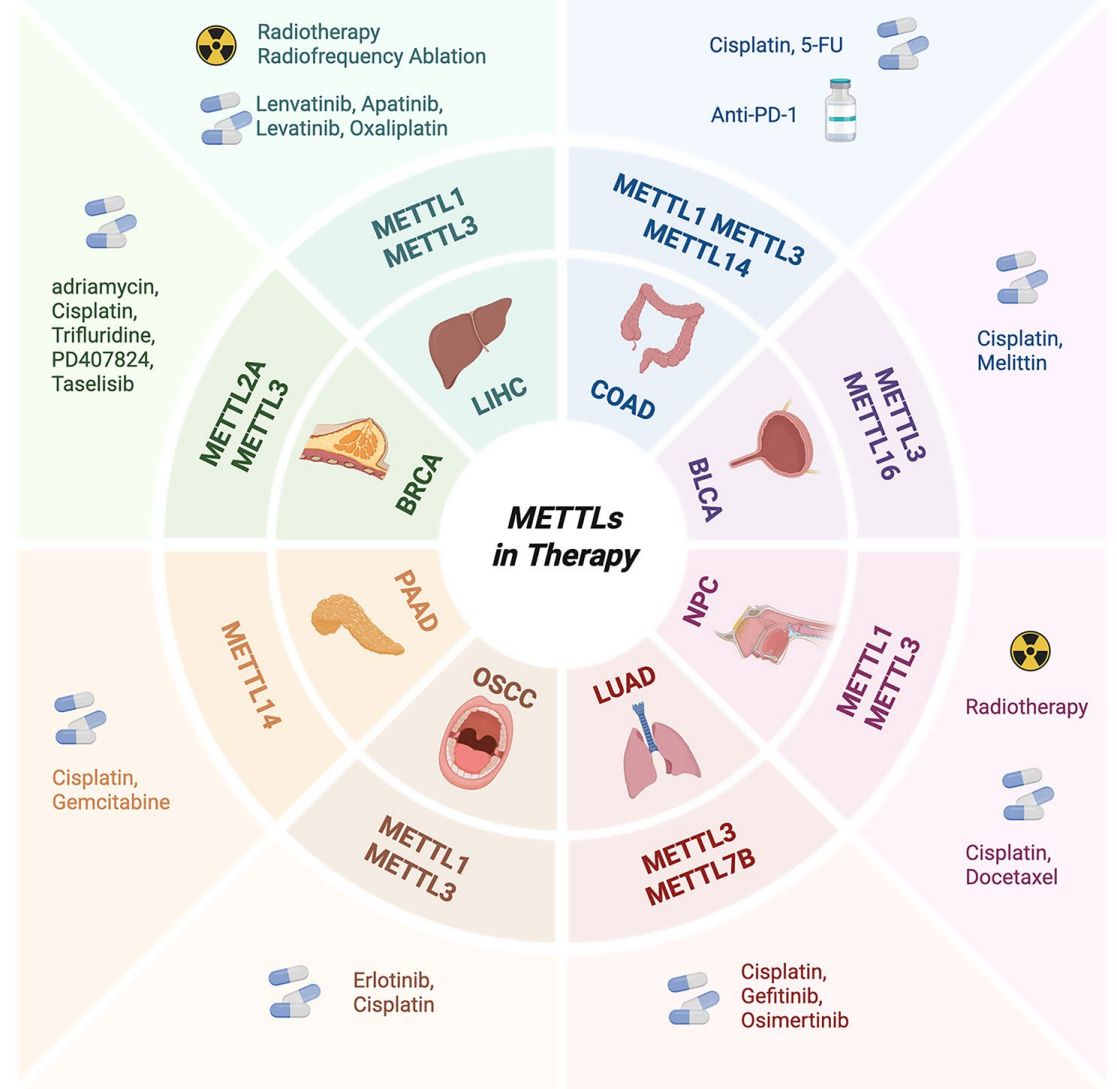
Summary of the prognostic role of METTL family members in diseases. Red, indicating that this METTL member is associated with a poor prognosis for this disease. Blue, indicating that this METTL member is associated with a better prognosis for this disease. The elevated expression of the majority of METTLs has been demonstrated to be associated with a poor disease prognosis. However, METTL7A and METTL17 are associated with a more favorable prognosis. METTL5, METTL14 and METTL16 have been observed to have different associations with prognosis in different diseases, which is consistent with their dual roles in the mechanism of tumorigenesis

‌Current research has demonstrated that various METTL proteins exhibit characteristic expression patterns in tumors, with their up/down-regulation frequently associated with critical prognostic indicators such as overall survival (OS) and progression-free survival (PFS). As shown in Table 1, METTL1, METTL3, METTL4, METTL5, METTL6 and METTL13 were associated with poor prognosis in various tumors including liver hepatocellular carcinoma (LIHC) [159, 160], breast invasive carcinoma (BRCA) [161–163], and lung adenocarcinoma (LUAD) [164]. Whereas METTL14, METTL16 and METTL17 represent different prognoses in different kinds of tumors [165–168] (Fig. 7).

**Table 1:** Basic functions and substrates of METTL family proteins

METTLS	ALIAS	SUBGROUP	SITE	SUBSTRATE
METTL1		DNA/RNA MTase	m7G	mRNA, tRNA
METTL2A		DNA/RNA MTase	m3C	tRNA
METTL2B		DNA/RNA MTase	m3C	tRNA
METTL3	MT-A70	DNA/RNA MTase	m6A	DNA, mRNA, tRNA, rRNA, lncRNA
METTL4		DNA/RNA MTase	m6A	mtDNA, mRNA, snRNA, miRNA, siRNA
METTL5		DNA/RNA MTase	m6A	rRNA
METTL6		DNA/RNA MTase	m3C	tRNA
METTL7	TMT1	small molecule MTase	sulfhydryl group	Exogenous sulfhydryl-containing compounds
METTL8		DNA/RNA MTase	m3C	DNA R-loop, mRNA, tRNA, lncRNA
METTL9		protein MTase	Histidine	S100A9, NDUFB3
METTL10	eEF1A-KMT2	protein MTase	Lysine	eEF1A
METTL11A	NTMT1, NRMT1	protein MTase	N-terminal X-P-K	free amino acid with X-P-K
METTL11B	NTMT2, NRMT2	protein MTase	N-terminal X-P-K	free amino acid with X-P-K
METTL12	CS-KMT	protein MTase	Lysine	Mitochondrial CS
METTL13	eEF1A-KNMT, FEAT	protein MTase	Lysine	eEF1A
METTL14		DNA/RNA MTase	m6A	DNA, mRNA, tRNA, rRNA, lncRNA, siRNA
METTL15		DNA/RNA MTase	m4C	mtRNA
METTL16		DNA/RNA MTase	m6A	mRNA, rRNA, lncRNA, mtRNA, snRNA
METTL17		small molecule MTase	F-S cluster	F-S cluster in mitochondrial ribosomal subunits
METTL18	C1orf156, AsTP2	protein MTase	Histidine	RPL3
METTL19	TRMT44	DNA/RNA MTase	NA	NA
METTL20	ETFβ-KMT	protein MTase	Lysine	ETFβ
METTL21A	HSPA-KMT	protein MTase	Lysine	HSPA1
METTL21B	eEF1A-KMT3	protein MTase	Lysine	eEF1A
METTL21C		protein MTase	Lysine	inconclusive
METTL21D	VCP-KMT	protein MTase	Lysine	VCP
METTL22		protein MTase	Lysine	KIN
METTL23		protein MTase	Arginine	GABPA

### The therapeutic potential of METTLs

**Fig. 8:**
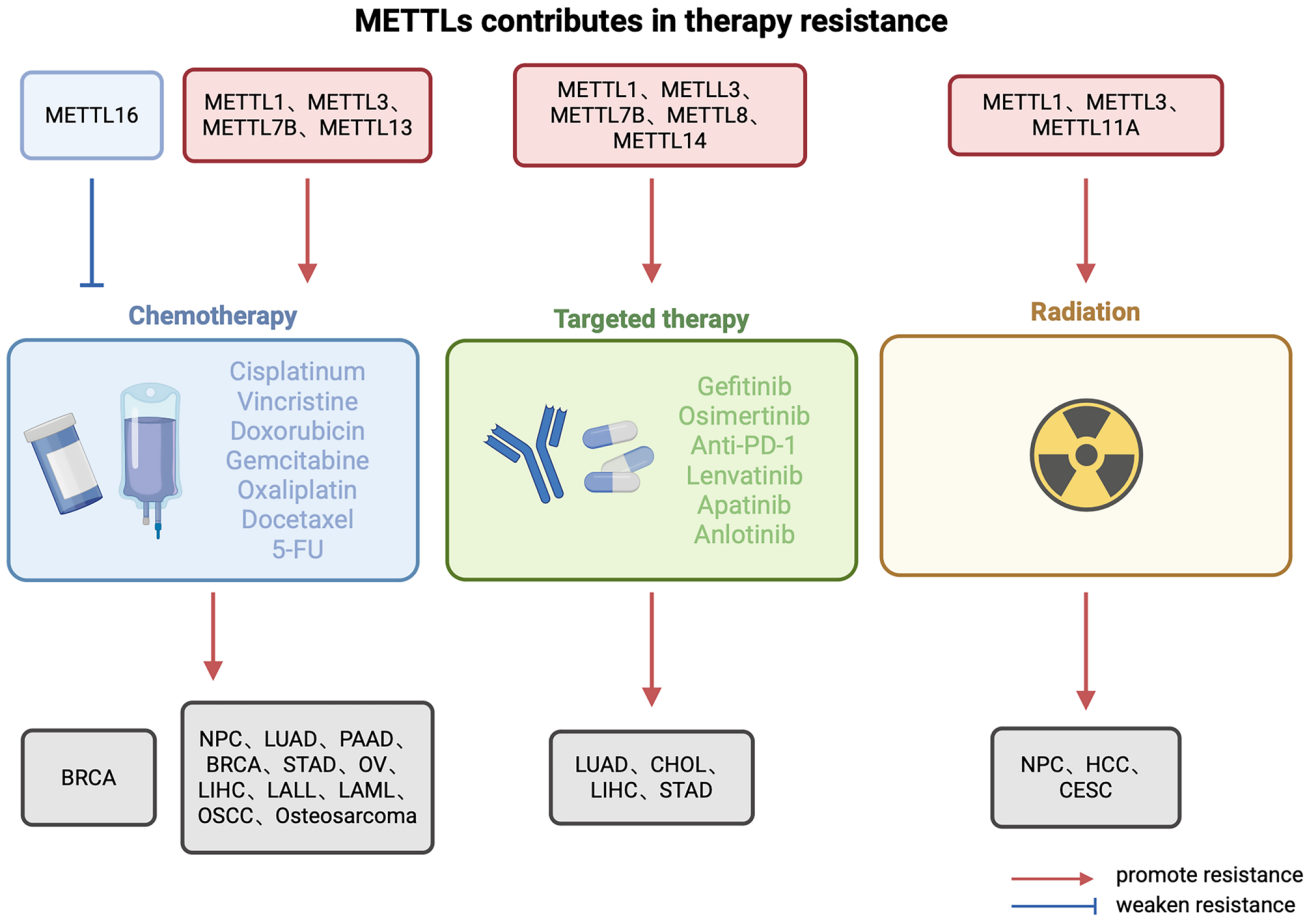
Summary of the role of METTLs on treatment resistance. METTL family is involved in tumor resistance to treatment. Despite the proliferation of anti-tumor therapies, some tumor cells still escape death by various mechanisms. The METTL family mainly plays a role in promoting the formation of resistance in chemotherapy, targeted therapy and radiotherapy. Red, suggested to play a role in promoting resistance formation. Blue, suggested to play a role in inhibiting resistance

Methyltransferase-like proteins have emerged as pivotal modulators of therapeutic response across diverse malignancies, presenting compelling opportunities for intervention strategies aimed at overcoming treatment resistance. METTL1 orchestrates multifaceted resistance mechanisms in hepatobiliary malignancies through precise epitranscriptomic reprogramming (Fig. 8). Elevated METTL1 expression correlates with diminished therapeutic efficacy through several distinct mechanisms in HCC. METTL1-catalyzed m7G tRNA modification selectively enhances translation of resistance-associated transcripts, attenuating responsiveness to lenvatinib—a first-line multikinase inhibitor for advanced HCC [185]. Following radiation therapy, METTL1 functions as a critical facilitator of non-homologous end-joining (NHEJ)-mediated DNA repair, thereby conferring radioresistance through enhanced damage resolution [186]. Building upon these findings, investigations into treatment modalities for early-stage HCC have revealed that radiofrequency ablation, despite being considered optimal, frequently results in treatment failure due to recurrence and metastasis. Mechanistic studies have unveiled the pivotal role of METTL1-mediated m^7^G tRNA modification in post-ablation recurrence through activation of the METTL1-m^7^G-SLUG/SNAIL axis following heat stress response. This molecular framework provides a rational basis for targeted intervention to mitigate post-ablation recurrence [187]. This resistance pattern extends beyond HCC to intrahepatic cholangiocarcinoma (ICC), where immunotherapy efficacy remains limited. In ICC models characterized by exceptionally poor responses to immune checkpoint inhibitors, dual inhibition of METTL1 and its downstream chemokine signaling substantially enhances anti-PD-1 efficacy, representing a promising immunotherapeutic strategy [188].

**Fig. 9:**
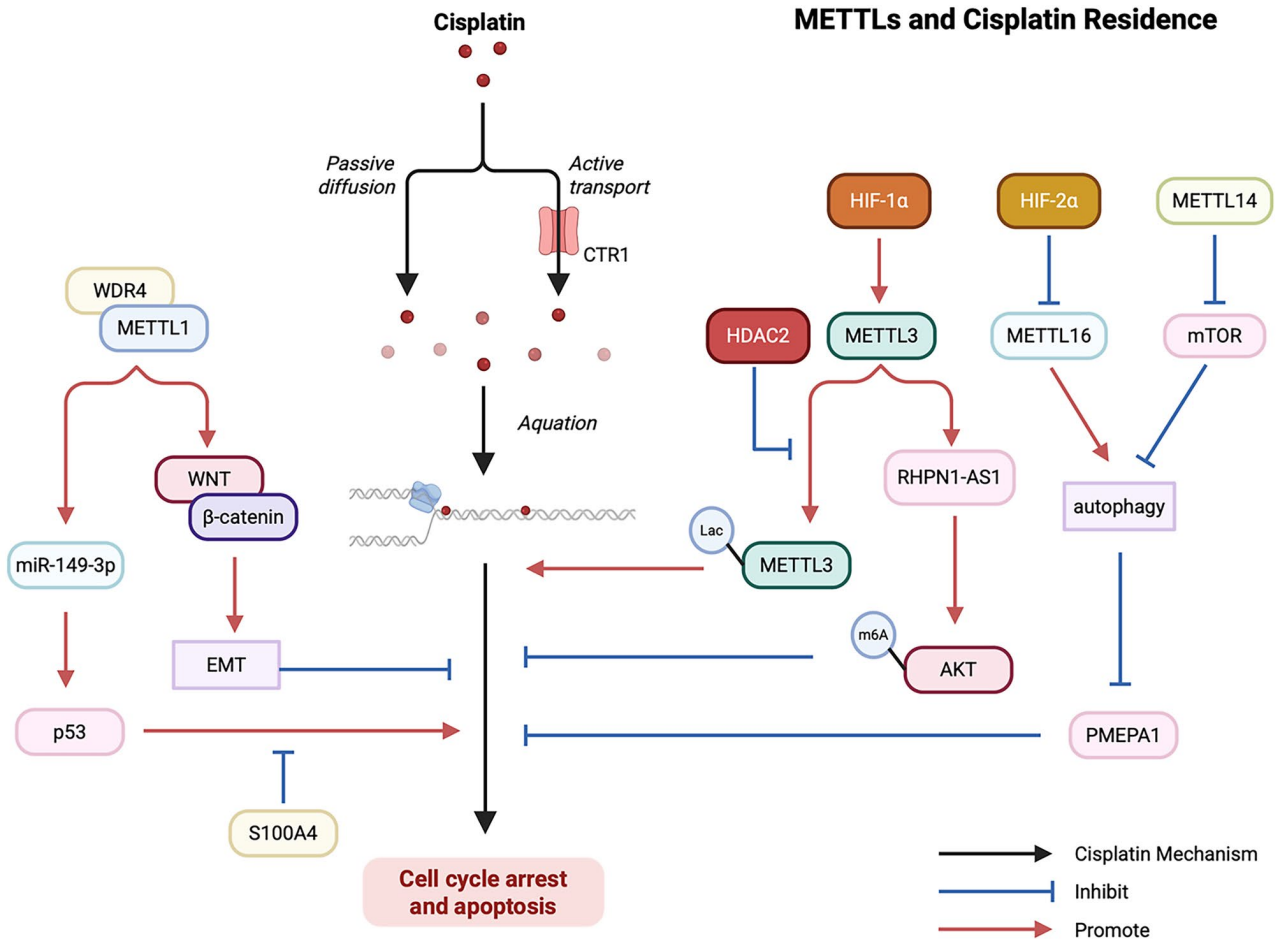
Diagram of METTL family members involved in the process of cisplatin resistance formation. Cisplatin is a chemotherapeutic agent that is frequently employed in the treatment of tumors. The action of METTL1 on the downstream WNT pathway and p53 inhibits and promotes cisplatin tumor killing, respectively. The de-lactivation of METTL3 and the activation of the AKT pathway promote the onset of cisplatin resistance. Both METTL14 and METTL16 are able to promote cisplatin resistance through autophagy-related pathways

Elevated expression of METTL1/WDR4 mediates m^7^G tRNA modification, activates the WNT/β-catenin pathway, and promotes EMT and chemoresistance to cisplatin and docetaxel in nasopharyngeal carcinoma (NPC) cells [169]. However, overexpression of METTL1 sensitize colorectal adenocarcinoma (COAD) cells to cisplatin by regulating the miR-149-3p/S100A4/p53 axis [170]. In addition, METTL1 promoted chemotherapy resistance to doxorubicin in Osteosarcoma [171], and mediated resistance to anlotinib in OSCC [172] (Fig. 9).

Numerous investigations have explored METTL3’s role in therapeutic resistance. In LIHC, METTL3 drives resistance to apatinib and Lenvatinib through m6A modification dependent pathway [173, 174]. METTL3 confers resistance to oxaliplatin by activating G6PD to enhance the pentose phosphate pathway [175] and leads to cisplatin resistance in OSCC, BRCA, ovarian serous cystadenocarcinoma (OV), and LUAD, suggesting the possibility of METTL3 inhibitor combined with platinum chemotherapy drugs [176–179]. Targeted inhibition of METTL3/LDHA axis can significantly improve the 5-FU sensitivity of COAD cells in vitro and in vivo [180]. Depletion of the methyltransferase METTL3 and METTL14 to suppress m^6^A mRNA modification enhanced the response of pMMR-MSI-L CRC and melanoma to anti-PD-1 therapy [181]. METTL3-mediated MALAT1/E2F1/AGR2 axis can promote Adriamycin resistance of BRCA [182]. METTL3 can also promote oxaliplatin resistance of gastric cancer (GC) cells by promoting the stability of PARP1 mRNA [183]. Similar to METTL1, METTL3 can also mediate the tolerance of NPC to radiotherapy [184]. METTL3 can inhibit the sensitivity of bladder cancer cells to Metilide treatment [185]. Down-regulation of METTL3 expression in bone marrow mesenchymal stem cells of acute myeloid leukemia (AML) induces an increase in AKT protein, leading to enhanced mesenchymal stem cell (MSC) adipogenesis, which contributes to chemotherapy resistance of AML cells [186]. In addition, two independent studies suggested that knockdown of METTL14 alone in pancreatic cancer enhanced sensitivity to cisplatin and gemcitabine [187, 188].

Beyond METTL1 and METTL3/14, several other METTL family members influence therapeutic responses. Drug sensitivity analysis suggested that Trifluridine, PD407824, and Taselisib were effective agents for METTL2A-positive BRCA patients [162]. METTL7B-mediated glycolysis regulates acute lymphoblastic leukemia cell proliferation and response to chemotherapy [189]. METTL7B inhibitor can be used to reverse the resistance of LUAD patients to tyrosine kinase inhibitor (TKIs) [12]. The high expression of METTL8 is negatively correlated with the sensitivity to Levatinib, but the specific mechanism is still unclear [190]. METTL11A helps drive tumorigenesis and serves as a marker of sensitivity to DNA-damaging chemotherapeutic agents or gamma irradiation [191]. METTL13-mediated CD44 mRNA decay in CRPC to promote tumor growth and metastasis, thereby promoting docetaxel treatment resistance [192]. METTL16 inhibits the proliferation and cisplatin resistance of bladder cancer by degrading PMEPA1 mRNA in the m6A manner through the autophagy pathway [193]. Collectively, these findings regarding METTL proteins in therapeutic resistance suggest promising combination treatment strategies, providing a conceptual and empirical foundation for future clinical translation.

### Clinical application of METTL inhibitors

Emerging pre-clinical and clinical data now position METTL-family inhibitors as a promising new class of anti-cancer agents. Early METTL3 inhibitors include cmpd2, sinefungin, and cpd-564. These compounds belong to the SAM analog class. While exhibiting some inhibitory activity, they demonstrate poor selectivity and cellular permeability [194, 195]. Subsequently, UZH1a was identified as a METTL3 inhibitor possessing an aminopyrimidine scaffold [196]. The STM2457 is the first METTL3 inhibitor to demonstrate antitumor activity and therapeutic efficacy in vivo. It has shown therapeutic potential in both myeloid leukemia and NSCLC, and can also improve immunotherapy outcomes based on PD-L1 upregulation [197, 198]. The STC-15 derivative of STM2457 is the first clinical candidate for an oral human formulation targeting METTL3 and has entered Phase I clinical trials (NCT 05584111). Compounds such as STM3006, CIDBA, quercetin, and eltrombopag have also been identified as METTL3 inhibitors; however, their antitumor effects in vivo have yet to be validated [199]. The development of inhibitors targeting other members of this family remains in very early stages. A nanomolar-level screening based on fluorescent RNA-methyltransferase probe technology identified three compounds—(S)-crizotinib, tanespimycin, and entacapone—as candidate METTL1 inhibitors. However, this study lacks corresponding biological functional validation [200]. A literature review reveals that current research on METTL family inhibitors is limited, with METTL3 being the most extensively studied member. Therefore, it is crucial to accelerate the development of METTL family molecular inhibitors, offering potential therapeutic options for cancer and metabolic diseases.

## Conclusions

As core regulators of nucleic acid modifications and protein PTMs modification, the METTL family orchestrates a dynamic and multidimensional regulatory network through methylation of RNAs and proteins, profoundly influencing fundamental biological processes and disease pathogenesis [3, 201]. In this review, we have systematically investigated its structure and function: from the conserved SAM-binding motifs to the diverse substrate recognition modules, from the m^6^A modification of mRNAs to the arginine methylation of proteins, and from the precise regulation of embryonic development to the complex intervention of tumors and neurodegenerative diseases, the METTL family has demonstrated its biological characteristics and “dual-use” functions. The METTL family embodies striking functional plasticity— “one enzyme, manifold roles”—and wields a context-dependent, double-edged sword in disease. This dual nature arises from a nexus of cell-type, substrate, and signaling cues that toggle METTL activity between tumor-suppressive and oncogenic outputs.

Tissue- and cell-type identity is the first gatekeeper: METTL abundance, the supply of their RNA/protein substrates, and the local stoichiometry of cofactors can differ by orders of magnitude across organs, so an identical mark can silence a tumor-suppressor in one context and activate an oncogene in another [202]. Layered on top are dynamic partnerships with “readers”, “erasers”, and scaffold proteins that assemble into context-specific editosomes, rewiring how the modification is deposited, interpreted, or removed and thereby redirecting downstream signaling networks [7].

Stoichiometry is the second dial: a sparse mark may stabilize an mRNA, while saturating methylation triggers its decay or reshapes the fold of a protein, switching enzyme kinetics on or off. These dosage effects are further tuned by extensive crosstalk-MAPK, hypoxia, or nutrient-sensing pathways can phosphorylate, acetylate, or relocalize METTLs themselves, turning the same catalytic domain from writer to scaffold or docking platform [203, 204]. Layered on top are developmental clocks, oxygen tension, oncogenic mutations, and cytokine storms that rewire the interactome within minutes, flipping the output from tumor suppression to invasion [205]. Together, this contextual circuitry explains why METTLs can act as guardians or saboteurs of the same process, and why precise molecular cartography—not bulk inhibition—will be required to safely exploit them as drug targets.

While sporadic reports have begun to map individual METTL members, there are still many research directions and hot spots for the METTL family in the future. (a) First of all is analysis of modification maps driven by single-cell and spatiotemporal omics. With the breakthrough of single-cell sequencing and spatial transcriptomics technology, future research will focus on mapping dynamic maps of methylation modifications with cell type specificity and subcellular compartment resolution. For example, analyzing how m^6^A modification mediated by METTL3 in local neuronal synapses regulates synaptic plasticity [206], or the “cross-talk” between immune cells and cancer cells in the tumor microenvironment by METTL1 [207]. Such studies are expected to reveal the spatiotemporal specificity of methylation modifications and provide molecular coordinates for precise intervention. (b) The second is substrate prediction and drug design enabled by artificial intelligence. There are still many unknowns in the molecular logic of substrate selectivity of the METTL family. Combining deep learning models (such as AlphaFold-Multimer) with high-throughput screening data, an “enzyme-substrate-microenvironment” interaction prediction system can be constructed in the future to crack the molecular code of METTL5 specifically targeting 18 S rRNA. In addition, generative AI will accelerate the virtual screening of new inhibitors, such as designing allosteric inhibitors that target the METTL3 phase separation interface (rather than the catalytic pocket) to enhance tissue selectivity [208]. (c) The third is innovation of targeted delivery and epigenome editing technology. The clinical application of METTL regulation is limited by delivery efficiency and off-target effects [209]. Tissue-specific delivery systems based on lipid nanoparticles or viral vectors (such as METTL14 activators targeting blood-brain barrier penetration for the treatment of Alzheimer’s disease) will become a research hotspot. At the same time, the fusion of CRISPR-dCas9 and epigenetic editing tools (such as dCas9-METTL3 fusion protein) can achieve site-specific RNA methylation editing, providing a new paradigm for the treatment of genetic diseases [210]. (d) The last but not the least, systematic integration of metabolic-epigenetic interaction networks. METTL family members (such as METTL16) participate in methylation homeostasis by regulating SAM metabolism, while metabolic reprogramming (including methionine cycle alterations in tumors) reciprocally influences their enzymatic activity [211]. Future research needs to integrate metabolomics and epigenetics to analyze the feedback loop of “metabolites-METTL activity-disease phenotype” and develop intervention strategies for regulating METTL function with small molecule metabolites (such as SAM analogs) [212].

Although research on the METTL family has made significant progress, its clinical transformation still faces core challenges [213]0.1. Precise regulation of the double-edged sword effect: METTL3 promotes cancer in hematological tumors, but suppresses cancer in some solid tumors, and biomarker-based patient stratification strategies need to be developed [214]; 2. Real-time monitoring of dynamic modification: develop probes that can image METTL activity in real time to evaluate treatment effects; 3. Systematic evaluation of long-term safety: the dynamic nature of methylation modification may trigger compensatory regulation, and organoid and human organ chip models need to be established for long-term toxicity testing. This revolution in epigenetics will not only redefine the paradigm of disease treatment, but will also reveal the ultimate code of dynamic regulation of life. Just as the discovery of the DNA double helix ushered in the era of molecular biology, deciphering the METTL family may represent a milestone in the transition of epigenetics toward “precision intelligent medicine.”

## Data Availability

Not applicable.

## Abbreviations

AML: Acute myeloid leukemia
METTL: Methyltransferase-like
m6A: N6-methyladenosine
m3C: 3-methylcytosine
m7G: N7-methylguanosine
PTMs: Protein post-translational modifications
SAM: S-adenosylmethionine
MTases: Methyltransferases
MTD: Methyltransferase domain
VCR: C-terminal vertebrate conserved region
m5C: 5-methylcytosine
NCBP: Nuclear cap-binding protein
QKI: Quaking protein
WDR4: METTL1/WD repeat structural domain 4
DALRD3: DALR anticodon binding domain containing 3
SerRS: Seryl-tRNA synthetase
lncRNA: Long-stranded non-coding RNAs
YTHDF: YTH domain proteins
HNRNPC: Heterogeneous nuclear ribonucleoprotein C
MACOM: METTL-associated complex
SCC: Spermatogonial stem cell
mtDNA: Mitochondrial DNA
m4C: N4-methylcytosine
mt-SSU: Mitochondrial small subunits
KMT: Lysine (K)-specific MTase
7BS: Hepta-β chain
eEF1A: Eukaryotic translation elongation factor 1α
eEF2: Eukaryotic translation elongation factor 2
CS: Citrate synthase
ETF: Electron transfer flavoprotein
HSPA: Heat Shock Protein Family A
VCP: Valine-containing protein
AARS1: 5-alanyl-tRNA synthetase 1
Arg: Arginine
PRMT: Protein Arginine Methyltransferase
RPL: Ribosomal protein large subunit
TMT: Thiol MTase
HDAC: Histone deacetylase
NSCLC: Non‑Small Cell Lung Cancer
CCND1: Cell cycle regulator cyclin D1
CDKN4: Cyclin-dependent kinase 4 inhibitor
HCC: Hepatocellular carcinoma
CRC: Colorectal Cancer
ACSL3: Acyl-CoA Synthetase Long Chain Family Member 3
NRF2: Nuclear factor erythroid 2-related factor 2
LDHA: Lactate dehydrogenase A
OSCC: Oral squamous cell carcinoma
TME: Tumor microenvironment
TAM: Tumor-associated macrophages
HDAC: Histone deacetylase
DM: Diabetes mellitus
T2D: Type 2 diabetes
NAFLD: Non-alcoholic fatty liver disease
FFA: Free fatty acid
OP: Osteoporosis
MI: Myocardial infarction
PRKCE: Protein kinase Cε
MI/R: MI/reperfusion
JAK2: Tetanus kinase 2
STAT3: Signal transducer and activator of transcription 3
EGR1: Early growth response protein 1
OTUD1: OTU Deubiquitinase 1
MAPK: Mitogen-activated protein kinase
CM: Cardiomyopathy
HF: Heart failure
NFκB: Nuclear Factor-κB
AS: Atherosclerosis
AD: Alzheimer’s disease
OA: Osteoarthritis
OS: Overall survival
PFS: Progression-free survival
LIHC: Liver hepatocellular carcinoma
BRCA: Breast invasive carcinoma
LUAD: Lung adenocarcinoma
NHEJ: Non-homologous end-joining
ICC: Intrahepatic cholangiocarcinoma
NPC: Nasopharyngeal carcinoma
COAD: Colorectal adenocarcinoma
OV: Ovarian serous cystadenocarcinoma
GC: Gastric cancer
AML: Acute myeloid leukemia
MSC: Mesenchymal stem cell
TKI: Tyrosine kinase inhibitor
